# Effect of drug metabolizing enzymes and transporters in Thai colorectal cancer patients treated with irinotecan-based chemotherapy

**DOI:** 10.1038/s41598-020-70351-0

**Published:** 2020-08-10

**Authors:** Chalirmporn Atasilp, Phichai Chansriwong, Ekaphop Sirachainan, Thanyanan Reungwetwattana, Suwannee Sirilerttrakul, Monpat Chamnanphon, Apichaya Puangpetch, Chonlaphat Sukasem

**Affiliations:** 1 Faculty of Medical Technology, Rangsit University, Pathum Thani, Thailand; 2 Division of Medical Oncology, Department of Medicine, Faculty of Medicine Ramathibodi Hospital, Mahidol University, Bangkok, Thailand; 3 Clinical Pharmacokinetics and Pharmacogenomics Research Unit, Department of Pharmacology, Faculty of Medicine, Chulalongkorn University, Bangkok, Thailand; 4 Division of Pharmacogenomics and Personalized Medicine, Department of Pathology, Faculty of Medicine Ramathibodi Hospital, Mahidol University, Bangkok, 10400 Thailand; 5 Laboratory for Pharmacogenomics, Clinical Pathology, Somdetch Phra Debharatana Medical Centre, Ramathibodi Hospital, Bangkok, Thailand

**Keywords:** Genetic testing, Genetic markers, Personalized medicine, Genetics research, Molecular medicine

## Abstract

Genetic polymorphisms in drug metabolizing enzymes and drug transporters may affect irinotecan toxicity. Although genetic polymorphisms have been shown to influence the irinotecan toxicity, data are limited in Thai population. Thus, the aim of this study was to assess the allele and genotype frequencies and the relationship between *CYP3A4/5*, *DPYD*, *UGT1A1*, *ABCB1*, and *ABCC2* genetic variations and irinotecan-induced toxicity in Thai colorectal cancer patients**.** One hundred and thirty-two patients were genotyped, and the effect of genetic variations on irinotecan-induced toxicity was assessed in 66 patients who received irinotecan-based chemotherapy**.** Allele frequencies of *ABCB1* c.1236C > T, *ABCB1* c.3435C > T, *ABCC2* c.3972C > T, *ABCG2* c.421C > A, CYP3A4*1B, CYP3A4*18, CYP3A5*3, DPYD*5, UGT1A1*28, and UGT1A1*6 were 0.67, 0.43, 0.23, 0.27, 0.01, 0.02, 0.64, 0.19, 0.16, and 0.09, respectively. DPYD*2A and *DPYD* c.1774C > T variants were not detected in our study population. The *ABCC2* c.3972C > T was significantly associated with grade 1–4 neutropenia (*P* < 0.012) at the first cycle. Patients carrying both UGT1A1*28 and *6 were significantly associated with severe neutropenia at the first (P < 0.001) and second (P = 0.017) cycles. In addition, patients carrying UG1A1*28 and *6 had significantly lower absolute neutrophil count (ANC) nadir at first (*P* < 0.001) and second (*P* = 0.001) cycles. This finding suggests that UGT1A1*28, *6, and *ABCC2* c.3972C > T might be an important predictor for irinotecan-induced severe neutropenia.

## Introduction

Irinotecan (CPT-11), a topoisomerase I inhibitor, is commonly used for the treatment of colorectal, gastric, and lung cancer. Although irinotecan is efficient, it causes severe neutropenia and diarrhea in 20–35% of the patients^1,2^. Irinotecan is metabolized by carboxylesterases (CESs) to form the primary pharmacologically active metabolite SN-38. SN-38, a topoisomerase I inhibitor, binds to and stabilizes the topoisomerase I-DNA complex preventing the re-ligation of DNA during replication and transcription, and subsequently results in double-stranded DNA breaks and apoptosis^3,4^. The SN-38 glucuronide (SN-38G), an inactive metabolite, is converted by uridine diphospho-glucuronosyltransferases (UGTs) in the liver and eliminated into bile by drug efflux transporters ABCB1, ABCC2, and ABCG2^5,6^.

Several studies have described the complex pharmacogenetics of irinotecan^7–9^. *UGT1A1* polymorphisms in promoter and coding regions are associated with reduced enzyme activity and accumulation of SN-38G which increases the toxicity of irinotecan. Previous studies reported that patients carrying UGT1A1*28 and *6 variants resulted in increased SN-38 activity leading to diarrhea and severe neutropenia^10,11^. *ABCB1* c.1236 C allele was significantly associated with grade 3/4 toxicities in metastatic colorectal cancer patients^12^, and *ABCB1* c.1236 T/T genotype was also associated with significantly increased exposure to irinotecan and its active metabolite SN-38 compared to those with heterozygous and wild-type^13^. *ABCB1* c.3435C > T altered expression levels and transport efficiency in vitro and in vivo^14,15^. *ABCC2* c.3972T/T genotype was related with higher areas under the plasma concentration–time curve (AUC) of irinotecan, (7-ethyl-10-[4-*N*-(5-aminopentanoic acid)-1-piperidino] carbonyloxycamptothecin) (APC) and SN-38G^16^. *ABCG2* c.421C > A variant results in lower protein expression and higher drug exposure^17^. Clinically, the combination of irinotecan and 5-fluorouracil is frequently used to treat colorectal cancer patients, and *DPYD* polymorphisms are related to 5-fluorouracil-induced severe neutropenia and diarrhea^18^.

Although there have been reports of the relationship between genetic polymorphisms and irinotecan induced-toxicity, there are few reports in Thai colorectal cancer patients. Therefore, the aim of this study was to extensively investigate the association between genetic polymorphisms in *CYP3A4/5*, *DPYD*, *UGT1A1*, *ABCB1*, *ABCC2*, and *ABCG2* and irinotecan induced-toxicity in cohort of Thai colorectal cancer patient.

## Methods

### Eligible patients

**Figure 1:**
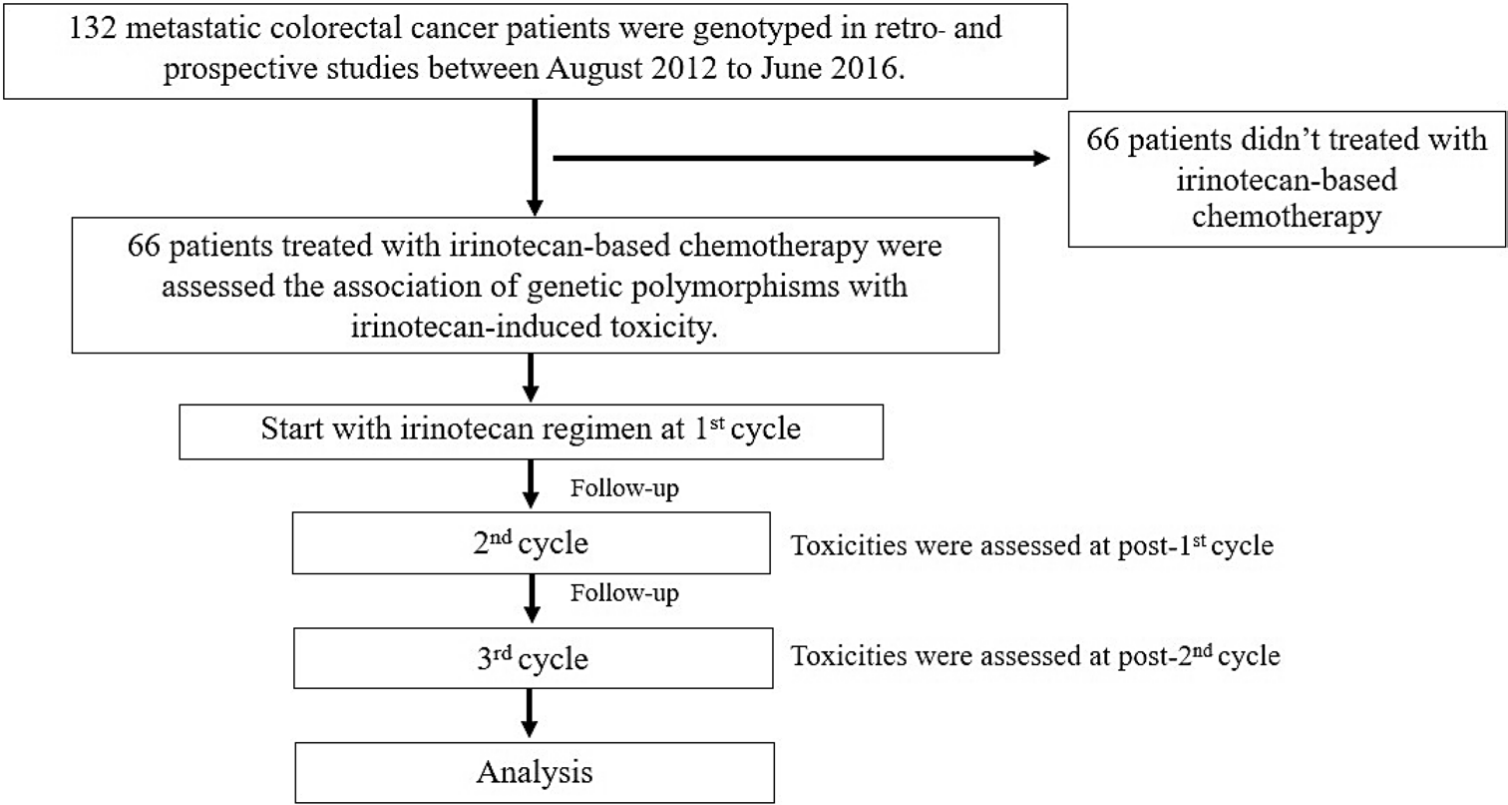
Flow chart for patient screening. A total of 132 metastatic colorectal cancer patients were genotyped for genetic polymorphisms and 66 patients who did not treated with irinotecan-based chemotherapy were excluded. Of the 66 patients treated with irinotecan-based chemotherapy were included in this analysis.

A total of 132 metastatic colorectal cancer patients who received chemotherapy were recruited in this retro- and prospective study between August 2012 and June 2016 from the Division of Cancer, Department of Medicine, Faculty of Medicine, Ramathibodi Hospital, Mahidol University, Thailand. The clinical eligibility criteria that was used to enroll patients were as follows: histologically or cytologically confirmed metastatic colorectal cancer; age at least 18 years; Eastern Cooperative Oncology Group (ECOG) performance status 0–2; life expectancy > 3 months; neutrophil count ≥ 1.5 × 10^9^/L; platelet count ≥ 8 × 10^10^/L; serum creatinine ≤ 1.25 upper limit normal (ULN); total bilirubin ≤ 1.25 ULN; alanine aminotransferase and aspartate aminotransferase ≤ 2.5 ULN. All patients had peripheral blood samples taken and complete clinical information (Supplementary Table 1). Sixty-six patients who were treated with irinotecan based-chemotherapy were analyzed for toxicity assessment. The flow chart for patient screening is shown in Fig. 1.

This study was approved by the Ethics Review Committee on Human Research of the Faculty of Medicine Ramathibodi Hospital, Mahidol University, Thailand (MURA2015/299) and conducted in accordance with the Declaration of Helsinki. The study protocol was clearly explained to all patients and informed consent was given before the study.

### Genotyping analysis

Peripheral blood was collected into an ethylenediaminetetraacetic acid (EDTA) tube, and genomic DNA was extracted using the MagNA Pure Compact System (Roche, Mannheim, Germany). DNA concentration was measured with a Thermo Scientific™ NanoDrop™ spectrophotometer and concentrations adjusted as recommended for each genotyping platforms. A total of 10 SNVs of CYP3A4*1B (c.-392A > G, Assay ID: C_1837671_50), CYP3A4*18 (c.878 T > C, C_27859823_20), CYP3A5*3 (c.6986A > G, C_26201809_30), DPYD*2A (IVS14 + 1G > A, C_30633851_20), DPYD*5 (c.1627A > G, C_1823316_20), *DPYD* c.1774C > T (C_90454263_10), *ABCB1* c.3435C > T (C_7586657_20), *ABCB1* c.1236C > T (C_758662_10), *ABCC2* c.3972C > T (C_11214910_20), and *ABCG2* c.421C > A (C_15854163_70) were genotyped by TaqMan^®^ Genotyping Assays (Applied Biosystems™, Carlsbad, CA, USA) according to the manufacturer’s instructions. An additional 2 variants, UGT1A1*28 (A(TA)7TAA) and *6 (c.211G > A), were genotyped by pyrosequencing (Qiagen, Japan) analysis according to a previously described method^19^.

### Drug administration

FOLFIRI regimen: Irinotecan 180 mg/m^2^, 90 min intravenous infusion on day 1; leucovorin (LV) 200 mg/m^2^ intravenous infusion on day 1; fluorouracil 400 mg/m^2^ intravenous bolus on day 1; fluorouracil 600 mg/m^2^ intravenous over the course of 46 h of continuous infusion; repeated every 2 weeks (28 patients).

FOLFIRI regimen plus cetuximab regimen: Cetuximab 400 mg/m^2^ intravenous infusion on day 1; Irinotecan 180 mg/m^2^, 90 min intravenous infusion on day 1; leucovorin (LV) 200 mg/m^2^ intravenous infusion on day 1; fluorouracil 400 mg/m^2^ intravenous bolus on day 1; fluorouracil 600 mg/m^2^ intravenous over the course of 46 h of continuous infusion; repeated every 2 weeks (seven patients).

FOLFIRI regimen plus bevacizumab regimen: Bevacizumab 5–10 mg/kg intravenous infusion once every 2 weeks; Irinotecan 180 mg/m^2^, 90 min intravenous infusion on day 1; leucovorin (LV) 200 mg/m^2^ intravenous infusion on day 1; fluorouracil 400 mg/m^2^ intravenous bolus on day 1; fluorouracil 600 mg/m^2^ intravenous over the course of 46 h of continuous infusion; repeated every 2 weeks (one patient).

Modified FOLFIRI regimen: Irinotecan 180 mg/m^2^, 90 min intravenous infusion on day 1; leucovorin (LV) 400 mg/m^2^ intravenous infusion on day 1; fluorouracil 400 mg/m^2^ intravenous bolus on day 1; fluorouracil 1,200 mg/m^2^ intravenous over the course of 46 h of continuous infusion; repeated every 2 weeks (18 patients).

Single irinotecan regimen: Irinotecan 100 mg/m^2^, 90 min intravenous infusion on day 1 (eight patients).

Irinotecan plus cetuximab or irinotecan plus capecitabine regimen: Irinotecan 100 mg/m^2^, 90 min intravenous infusion on day 1; cetuximab 100–130 mg/m^2^ intravenous infusion on day 1 or irinotecan 100 mg/m^2^, 90 min intravenous infusion on day 1; capecitabine 1,000 mg/m^2^ (four patients).

### Toxicity criteria

Toxicity was assessed at first and second cycles of treatment according to National Cancer Institute Common Toxicity Criteria for Adverse Events, version 5.0. Grade 3–4 toxicity was considered as severe toxicity.

### Statistical analysis

Deviation from Hardy–Weinberg equilibrium was assessed using Fisher’s exact and chi-square test. Allele and genotype frequencies were determined by direct counting. Comparisons of allele and genotype frequencies and grades of toxicity were performed using the χ^2^ test. Mann–Whitney U test was performed according to difference of genetic groups and nonparametric data [absolute neutrophil count (ANC) nadir and ANC ratio]. Logistic regression analysis was performed to assess univariate and multivariate relationships genetic polymorphisms, and other parameters. All statistics were calculated using SPSS version 18 (SPSS Inc., Chicago, IL, USA) and differences were significant when *P* values were < 0.05.

## Results

### Clinical characteristics and genotyping data

A total of 132 metastatic colorectal cancer patients were genotyped for CYP3A4*1B, CYP3A4*18, CYP3A5*3, DPYD*2A, DPYD*5, *DPYD* c.1774C > T, UGT1A1*28, UGT1A1*6, ABCB1 c.1236C > T, *ABCB1* c.3435C > T, *ABCC2* c.3972C > T, and *ABCG2* c.421C > A. The genotype and allele frequencies are shown in Table 1. The most prevalent alleles were *ABCB1* c.1236C > T (0.67), CYP3A5*3 (0.64), and *ABCB1* c.3435C > T (0.43), respectively. DPYD*2A and *DPYD* c.1774C > T were not detected in our samples.

**Table 1:** Genotype and allele frequencies of 132 metastatic colorectal cancer patients.

Gene	Polymorphisms	Genotype frequency (%)			Allele frequency	
		W/W	W/V	V/V	W	V
*ABCB1*	c.1236C > T (rs1128503)	12 (9.1)	64 (48.5)	56 (42.4)	0.33	0.67
	c.3435C > T (rs1045642)	45 (34.1)	60 (45.5)	27 (20.5)	0.57	0.43
*ABCC2*	c.3972C > T (rs3740066)	75 (56.8)	52 (39.4)	5 (3.8)	0.77	0.23
*ABCG2*	c.421C > A (rs2231142)	72 (54.6)	49 (37.1)	11 (8.3)	0.73	0.27
*CYP3A4*	*1B (c.-392A > G, rs2740574)	131 (99.2)	1 (0.8)	0 (0)	0.99	0.01
	*18 (c.878 T > C, rs28371759)	127 (96.2)	5 (3.8)	0 (0)	0.98	0.02
*CYP3A5*	*3 (c.6986A > G, rs776746)	18 (13.6)	59 (44.7)	55 (41.7)	0.36	0.64
*DPYD*	*2A (IVS14 + 1G > A, rs3918290)	132 (100)	0 (0)	0 (0)	1.00	0.00
	*5 (c.1627A > G, rs1801159)	85 (64.4)	44 (33.3)	3 (2.3)	0.81	0.19
	c.1774C > T (rs59086055)	132 (100)	0 (0)	0 (0)	1.00	0.00
*UGT1A1*	*28 (A(TA)7TAA, rs3064744)	94 (71.2)	35 (26.5)	3 (2.3)	0.84	0.16
	*6 (c.211G > A, rs4148323)	107 (81.1)	25 (18.9)	0 (0)	0.91	0.09

*W* wild type, *V* variant.

Sixty-six patients with metastatic colorectal cancer receiving an irinotecan-based regimen were enrolled for association analysis. Their clinical characteristics are summarized in Table 2. The average age of the 66 patients was 62 years (range 25–74) with 42 (63.6%) male and 24 (36.4%) female. Most patients showed an ECOG performance status of zero. The most common site of disease was the rectum. The liver was the dominant site for metastases. There were no statistically significant differences between clinical characteristics and hematological toxicity including neutropenia, leucopenia, thrombocytopenia, and anemia (data not shown).

**Table 2:** Clinical characteristics of 66 colorectal cancer patients.

Characteristics	Number of patients (%)
Age (years), mean ± SD	62 ± 12
**Gender**	
Male	42 (63.6)
Female	24 (36.4)
**ECOG performance status**	
0	35 (53)
1	26 (39.4)
2	5 (7.6)
**Site of disease**	
Rectum	30 (45.5)
Sigmoid	16 (24.2)
Right side	8 (12.1)
Rectosigmoid	5 (7.6)
Left side	5 (7.6)
Transverse	2 (3)
**Sites of metastases**	
Liver	54 (59.4)
Lung	31 (34)
Others	6 (6.6)
**Histopathology type**	
Well differentiated	16 (24.2)
Moderately differentiated	49 (74.2)
Poorly differentiated	1 (1.6)
**Line of treatment**	
First line	11 (16.7)
Second line	42 (63.6)
Third line	13 (19.7)
**Treatment regimen**	
FOLFIRI	28 (42.4)
Modified FOLFIRI	18 (27.3)
Irinotecan	8 (12.2)
FOLFIRI + cetuximab	7 (10.6)
Irinotecan + capecitabine	3 (4.5)
FOLFIRI + bevacizumab	1 (1.5)
Irinotecan + cetuximab	1 (1.5)

*ECOG* Eastern Cooperative Oncology Group.

### Association between genetic polymorphisms and irinotecan-induced neutropenia

The association analysis is summarized in Table 3. At the first cycle of treatment, *ABCC2* c.3972C > T was significantly associated with all grades neutropenia [grade 1–4 neutropenia; odds ratio (OR) 3, 95% confidence intervals (CI) 1.3–7; *P* < 0.012]. In addition, UGT1A1*6 was significantly associated with grade 1–4 and severe neutropenia (grade 3–4) (OR 20.3, 95% CI 4.3–95.6; *P* < 0.001, and OR 4, 95% CI 1.2–13; *P* < 0.026, respectively). Although there were no significant differences between grade 3–4 neutropenia and patients with UGT1A1*28, the incidence of severe neutropenia in patients with hetero- and homozygous *28 was higher than patients with homozygous wild type, (OR 2.7, 95% CI 0.8–8.8; *P* = 0.087).

**Table 3:** Genetic polymorphisms associated with neutropenia in first and second cycles (N = 66).

Gene	Genotype	N	Toxicity (neutropenia)							
			First cycle				Second cycle			
			Grade 1–4^a^ n (%)	*P*	Grade 3–4^b^ n (%)	*P*	Grade 1–4^a^ n (%)	*P*	Grade 3–4^b^ n (%)	*P*
***ABCB1***										
c.1236C > T	C/C	9	4 (44.4)	0.946	1 (11.1)	1.000	4 (44.4)	1.000	1 (11.1)	1.000
	C/T + T/T	57	26 (45.6)		9 (15.8)		26 (45.6)		9 (15.8)	
c.3435C > T	C/C	23	9 (39.1)	0.407	2 (8.7)	0.352	9 (39.1)	0.407	1 (4.3)	0.070
	C/T + T/T	43	21 (48.8)		8 (18.6)		21 (48.9)		9 (20.9)	
***ABCC2***										
c.3972C > T	C/C	38	13 (34.2)	0.012^†^	6 (15.8)	0.841	15 (39.5)	0.179	5 (13.2)	0.582
	C/T + T/T	28	17 (60.7)		4 (14.3)		15 (53.6)		5 (17.9)	
***ABCG2***										
c.421C > A	C/C	36	17 (47.2)	0.711	5 (13.9)	0.717	15 (41.7)	0.428	5 (13.)	0.717
	C/A + A/A	30	13 (43.3)		5 (16.7)		15 (50)		5 (16.7)	
***CYP3A4***										
*1B (c.-392A > G)	A/A	66	30 (45.5)	ND	10 (15.2)	ND	30 (48.5)	ND	10 (15.2)	ND
*18 (c.878T > C)	T/T	65	30 (46.2)	ND	10 (15.4)	ND	30 (46.2)	ND	10 (15.4)	ND
	T/C + C/C	1	0 (0)	ND	0 (0)	ND	0 (0)	ND	0 (0)	ND
***CYP3A5***										
*3 (c.6986A > G)	A/A	7	3 (42.9)	1.000	0 (0)	0.596	3 (42.9)	1.000	1 (14.3)	1.000
	A/G + G/G	59	27 (45.8)		10 (16.9)		27 (45.8)		9 (15.3)	
***DPYD***										
c.1774C > T	C/C	66	30 (45.5)	ND	10 (15.2)	ND	30 (48.5)	1.000	10 (15.2)	ND
*2A (IVS14 + 1G > A)	G/G	66	30 (45.5)	ND	10 (15.2)	ND	30 (48.5)	1.000	10 (15.2)	ND
*5 (c.1627A > G)	A/A	41	20 (48.8)	0.401	7 (17.1)	0.491	19 (46.3)	0.836	8 (19.5)	0.106
	A/G + G/G	25	10 (40)		3 (12)		11 (44)		2 (8)	
***UGT1A1***										
*28 (A(TA)7TAA)	TA6/TA6	51	21 (41.2)	0.102	6 (11.8)	0.087	20 (39.2)	0.017^†^	8 (15.7)	1.000
	TA6/TA7 + TA7/TA7	15	9 (60)		4 (26.7)		10 (66.7)		2 (13.3)	
*6 (c.211G > A)	G/G	54	19 (35.2)	< 0.001^†^	6 (1.9)	0.026^†^	19 (35.1)	< 0.001^†^	4 (7.4)	< 0.001^†^
	G/A + A/A	12	11 (91.7)		4 (33.3)		11 (91.7)		6 (50)	
***UGT1A1*genotype**										
Homozygous wild type	*1/*1	40	10 (25)		2 (5)		10 (25)		3 (7.5)	
Heterozygous variant	*1/*28,*1/*6	24	19 (79.2)	< 0.001^†^	8 (33.3)	0.002^†^	18 (75)	< 0.001^†^	6 (25)	0.016^†^
Homozygous variant	*28/*28, *28/*6	2	1 (50)		1 (50)		2 (100)		1 (50)	

*ND* not determine.

^†^*p* value < 0.05 was *considered *statistically* significant*.

^a^Grade 1–4 was considered as toxicity.

^b^Grade 3–4 was considered as severe toxicity.

At the second cycle, an association was observed between UGT1A1*28 and grade 1–4 neutropenia (OR 3.1, 95% CI 1.2–7.97; *P* = 0.017). Similarly, UGT1A1*6 was significantly associated with grade 1–4 (OR 20.3, 95% CI 4.3–95.6; *P* < 0.001) and severe neutropenia (OR 12.5, 95% CI 3.4–45.7; *P* < 0.001).

The combination of UGT1A1*28 and *6 showed a significant increased risk for all grades of neutropenia (*P* < 0.001) and severe neutropenia (*P* = 0.002) at first cycle. Similarly in the second cycle, patients with hetero- and homozygous variant had a high incidence of all grades of neutropenia (*P* < 0.001) and severe neutropenia (*P* = 0.016).

A multivariate logistic regression analysis was performed to analyze the influence of CYP3A4*1B, CYP3A4*18, CYP3A5*3, DPYD*2A, DPYD*5, *DPYD* c.1774C > T, UGT1A1*28, UGT1A1*6, *ABCB1* c.1236C > T, *ABCB1* c.3435C > T, *ABCC2* c.3972C > T, and *ABCG2* c.421C > A on neutropenia (all grades and severe neutropenia) at first and second cycles. The result showed that *ABCC2* c.3972C > T was significantly associated with grade 1–4 neutropenia (*P* = 0.015). In the second cycle, we found patients with UGT1A1*28 were at significant increased risk for grade 1–4 neutropenia compared with wild type patients (*P* = 0.011). Moreover, patients with UGT1A1*6 were at significantly increased risk for grades 1–4 and severe neutropenia compared with wild type patients (*P* = 0.002, *P* = 0.001, respectively), as shown in Table 4.

**Table 4:** Multivariate logistic regression analysis to analyze the factors affecting neutropenia at first and second cycles.

Factors	First cycle			Second cycle					
	Grade 1–4 neutropenia^a^			Grade 1–4 neutropenia^a^			Grade 3–4 neutropenia^b^		
	Exp (B)	95% CI	*P* value	Exp (B)	95% CI	*P* value	Exp (B)	95% CI	*P* value
*ABCC2* 3972C > T	5.06	1.38–18.63	0.015^†^						
*UGT1A1* *28 (A(TA)7TAA)				5.44	1.48–20.02	0.011^†^			
UGT1A1*6 (211G > A)				30.67	3.51–268.36	0.002^†^	12.50	2.73–57.29	0.001^†^

*Exp* exponential, *95% CI* 95% confidence interval.

^†^*p* value < 0.05 was *considered *statistically* significant.*

^a^Grade 1–4 was considered as toxicity.

^b^Grade 3–4 was considered as severe toxicity.

**Figure 2:**
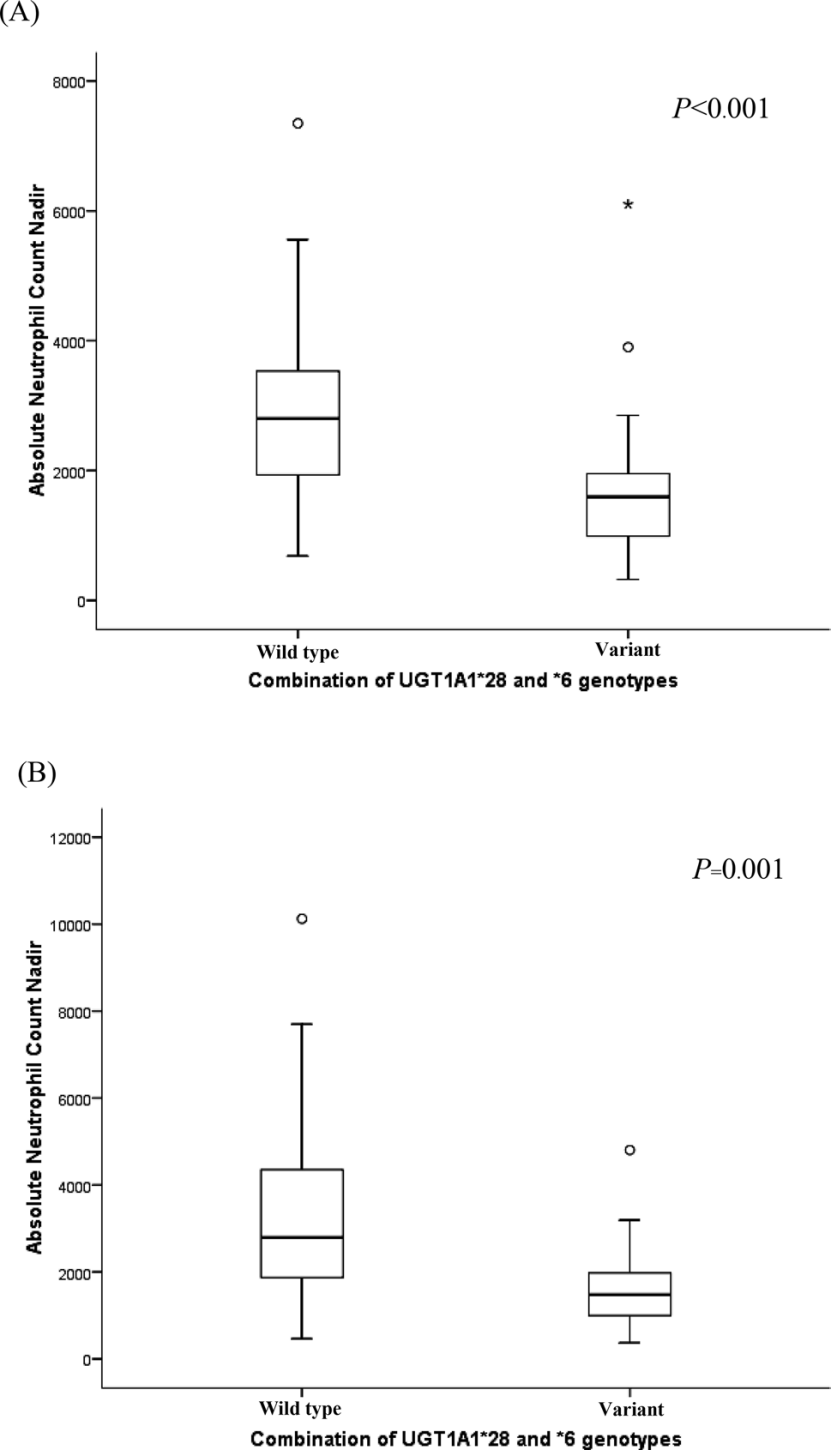
Association of combined *UGT1A1* genotype (*28 and *6) with absolute neutrophil count nadir (/mm^3^) at first cycle and second cycle. (**A**) At first cycle, (**B**) at second cycle.

The association of genetic polymorphisms and absolute neutrophil count (ANC) nadir was also assessed at the first and second cycle. Regarding UGT1A1*6, the ANC nadir of G/A was significantly lower than A/A in both first (1,600:2,560.7/mm^3^, *P* = 0.004) and second (1,201.8:2,379.8/mm^3^, *P* < 0.001) cycles. Hetero- and homozygous UGT1A1*28 or *6 carriers showed decreased ANC nadir compared to wild type carriers at first (1,595.9:2,894.1/mm^3^, *P* < 0.001) and second 1,528.4:2,793/mm^3^, *P* = 0.001) cycles (Fig. 2).

**Figure 3:**
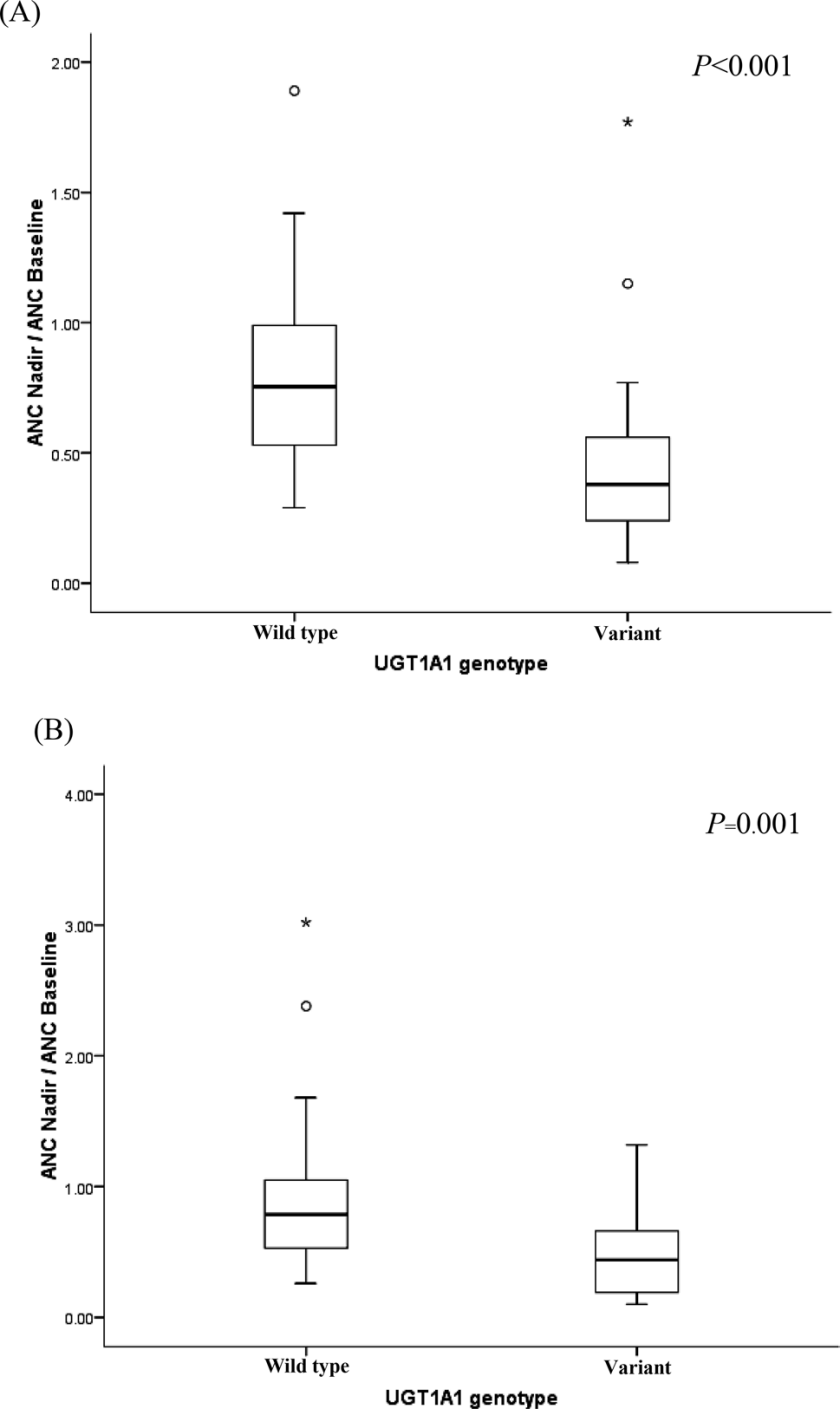
Association of combined *UGT1A1* genotype (*28 and *6) with absolute count neutrophil (ANC) nadir to the ANC baseline (pretreatment) at first cycle and second cycles. (**A**) At first cycle, (**B**) at second cycle.

Using ANC ratio (ANC nadir to ANC baseline), patients carrying a variant of *UGT1A1* had lower ANC ratio at first (0.41:0.79; *P* < 0.001) and second (0.45:0.79; *P* = 0.001) cycles, (Fig. 3). ANC ratio in patients with *UGT1A1* c.211 G/A was significantly lower than G/G at the first (0.4:0.6, *P* = 0.020) and second (0.3:0.7, *P* = 0.010) cycles, and UGT1A1*28 was significantly associated with decreased ANC ratio (variant: wild type; 0.47:0.72, *P* = 0.047) at the first cycle.

## Discussion

In this study, the association between irinotecan-induced toxicity and pharmacogenetics of drug metabolizing enzymes and drug transporters was investigated. Our results showed that combined analysis of UGT1A1*28 and *6 polymorphisms and *ABCC2* c.3972C > T were closely related with neutropenia toxicity in Thai colorectal cancer patients.

The UGT1A1*28 allele is the most important risk factor for severe neutropenia or diarrhea. In 2005, the U.S. Food and Drug Administration (FDA) informed that patients with homozygous UGT1A1*28 are at increased risk of severe neutropenia following initiation of irinotecan treatment^20^. Several studies have investigated the relationship of UGT1A1*28 and severe neutropenia and diarrhea during irinotecan treatment^21–23^. Wang et al*.*^24^ reported a significantly high risk for grade 3–4 leukopenia and neutropenia in patients carrying heterozygous UGT1A1*28 compared to homozygous wild type patients. Similarly, Rouits et al*.*^25^ reported that patients carrying the homozygous or heterozygous UGT1A1*28 had significantly higher risk of neutropenia than those with UGT1A1*1. In this study, although there were no significant differences between grade 3–4 neutropenia and patients with UGT1A1*28, the incidence of severe neutropenia in patients with hetero- and homozygous *28 was higher than patients with homozygous wild type at the first cycle (OR 2.7, 95% CI 0.8–8.8; *P* = 0.087). At the second cycle, patient carried UGT1A1*28 was a significantly higher risk of neutropenia than patients with homozygous wild type (OR 3.1, 95% CI 1.2–7.97; *P* = 0.017). A multivariate analysis was performed to analyze the influence of UGT1A1*28. This result shown that patients with UGT1A1*28 was at significant increased risk for grade 1–4 neutropenia compared with wild type patients (*P* = 0.011) in second cycle. Using ANC ratio, patient carried heterozygous and homozygous UGT1A1*28 had lower ANC nadir and ANC ratio than wild type at first cycle (*P* = 0.047). These results shown an increased risk of neutropenia in patient with UGT1A1*28 genotype.

The prevalence of UGT1A1*6 in Asian is higher than Caucasian population. The UGT1A1*6 polymorphism is the most common allele that is correlated with reduced SN-38 glucuronidation activity and drug toxicity^26^. Han et al*.*^27^ demonstrated that UGT1A1*6/*6 was significantly associated with higher SN-38 AUC and may increase the risk for toxicities. Onoue et al*.*^28^ performed a prospective study of 135 Japanese cancer patients treated with irinotecan, and found that severe neutropenia was highly correlated with homozygous UGT1A1*6 in a multiple logistic regression analysis. Similarly, UGT1A1*6 was significantly associated with grade 1–4 and severe neutropenia at the first and second cycles in this study. In contrast, there were significant differences between grade 1–4 neutropenia and patients with UGT1A1*28 at the second cycle. However, *UGT1A1* genotype was associated with an increased risk of grade 1–4 and severe neutropenia at the first and second cycle. Similar to the study by Yang et al.^29^, UGT1A1*28 and *6 were significantly associated with higher incidence of grade 3–4 neutropenia. A meta-analysis by Han et al.^30^ found that Asian cancer patients with UGT1A1*28 and *6 are at increased risk of irinotecan-induced neutropenia. Moreover, we also found an association between UGT1A1*28 or/and UGT1A1*6 and ANC nadir. This revealed that patients carrying variant of *UGT1A1* genotype had a significantly lower ANC nadir in the first and second cycle. Moriya et al*.*^31^ reported that ANC nadir in patients carrying UGT1A1*6/*28, *6/*6 were significantly lower compared with those with *1/*1.

ABCC2 protein is expressed in liver, kidney, and small intestine, and also plays a primary role in biliary excretion of irinotecan and its metabolites^32,33^. Interestingly, our result suggested that *ABCC2* c.3972C > T is associated with grade 1–4 neutropenia at the first cycle. Multivariate analysis indicated that *ABCC2* c.3972C > T is a risk factor for the occurrence of grade 1–4 neutropenia at the first cycle in patients who receive irinotecan-based chemotherapy. Innocenti et al.^16^ reported that *ABCC2* c.3972T/T genotype correlates with higher AUC of irinotecan, APC, and SN-38G. This result suggests that *ABCC2* c.3972C > T is associated with decreased hepatobiliary excretion of irinotecan and its metabolites.

*ABCB1* c.3435C > T is associated with significantly lower AUC SN-38G levels, and homozygous *ABCB1* c.3435T/T may be related to higher P-glycoprotein (MDR1) activity^34^. However, *ABCB1* c.3435C > T was not associated with irinotecan induced severe neutropenia and diarrhea in Chinese cancer patients who received irinotecan chemotherapy^35^. Cote et al.^36^ reported that no statistically significant difference was found in *ABCB1* c.3435C > T polymorphism and occurrence of severe hematologic toxicity or severe neutropenia.

The *ABCB1* c.1236C > T has been reported to be associated with increased AUC of irinotecan and SN-38 in Caucasian cancer patients^13^, and *ABCB1* c.1236T/T had significantly higher plasma irinotecan and SN-38 concentrations than C/C or C/T. However, Han et al.^34^ reported that no significant effect of *ABCB1* c.1236C > T on irinotecan or its metabolites concentrations. Han et al.^27^ reported that no significant association between *ABCB1* c.1236C > T and severe neutropenia and diarrhea was observed. In vitro studies have shown that ABCG2, an efflux drug transporter, had a higher affinity with SN-38 and SN-38G^37^, and *ABCG2* c.421C > A is related with reduced expression of ABCG2 protein and transporter activity. However, de Jong et al*.*^38^ reported that no significant changes in irinotecan pharmacokinetics relative to the *ABCG2* c.421C > A in Caucasian cancer patients. *ABCG2* variants had no effect on SN-38 exposure or ANC nadir in 78 irinotecan-treated patients^39^.

Irinotecan is converted by CYP3A4/5 to APC metabolite in the liver, and correlation between these genes and irinotecan induced-toxicity found in this study may be due to low allele frequency of CYP3A4*1B and *18 in sampled population^40^. Similarly, Han et al*.*^34^ did not find any significant association between CYP3A5*3 and toxicity.

The 5-FU-based regimen may cause neutropenia, however, over 80% of 5-FU is metabolized by dihydropyrimidine dehydrogenase (DPD) in the liver^41^. *DPYD* variants may be related to severe 5-FU-associated toxicities. DPYD*2A and c.1774 C > T variants were not found in this study. Even though, the variant allele of DPYD*5 had a frequency of 0.19, there was no association between DPYD*5 and hematological toxicities.

A retrospective study design and small sample size are limitations of this study. A prospective study involving larger numbers of patients should confirm our study hypothesis. Secondly, rare genetic variants and multiple genes play a role in the irinotecan pathway. Those variants were not considered in our study. Lastly, non-hematologic toxicity (especially severe diarrhea) was not assesses in our study.

In conclusion, combination of UGT1A1*28 and *6 and *ABCC2* c.3972C > T genotype are associated with the occurrence of grade 1–4 and severe neutropenia in Thai patients with metastatic colorectal cancer who receive irinotecan-based chemotherapy. Our findings suggest that *UGT1A1* genotype and *ABCC2* c.3972C > T might be an important predictor for irinotecan induced-toxicity.

## Supplementary information

Supplementary Table 1.
