# Supplementary material for: Effect of drug metabolizing enzymes and transporters in Thai colorectal cancer patients treated with irinotecan-based chemotherapy
Source: Sci Rep. 2020 Aug 10;10:13486. doi: 10.1038/s41598-020-70351-0 (PMC7417535; doi:10.1038/s41598-020-70351-0)
Supplement: Supplementary file 1 — Supplementary Table 1. [file 41598_2020_70351_MOESM1_ESM.pdf]

Effect of drug metabolizing enzymes and transporters in Thai colorectal cancer patients treated with irinotecan-based chemotherapy

Chalirmporn Atasilp[1], Pichai Chansriwong[2], Ekapob Sirachainan[2], Thanyanan Reungwetwattana[2], Suwannee Sirilerttrakul[2], Monpat Chamnanphon[3], Apichaya Puangpetch[4,5] & Chonlaphat Sukasem[4,5*]

[1]Faculty of Medical Technology, Rangsit University, Pathum Thani, Thailand
[2]Division of Medical Oncology, Department of Medicine, Faculty of Medicine Ramathibodi Hospital, Mahidol University, Bangkok, Thailand
[3]Clinical Pharmacokinetics and Pharmacogenomics Research Unit, Department of Pharmacology, Faculty of Medicine, Chulalongkorn University, Bangkok, Thailand
[4]Division of Pharmacogenomics and Personalized Medicine, Department of Pathology, Faculty of Medicine Ramathibodi Hospital, Mahidol University, Bangkok, Thailand
[5]Laboratory for Pharmacogenomics, Clinical Pathology, Somdetch Phra Debharatana Medical Centre, Ramathibodi Hospital, Bangkok, Thailand

[*]**Correspondence to: Chonlaphat Sukasem, B.Pharm., Ph. D**

Division of Pharmacogenomics and Personalized Medicine, Department of Pathology, Faculty of Medicine, Ramathibodi Hospital, Mahidol University, Bangkok, Thailand, 10400, Email: chonlaphat.suk@mahidol.ac.th

Telephone: (+66)-2-200-4330

Fax: (+66)-2-200-4332

Characteristics	Number of patients (%)
**Age (years), range**	62 (53-71)
**Gender**	
Male	78 (60.0)
Female	54 (40.0)
**Site of disease**	
Rectum	55 (41.7)
Sigmoid	38 (28.8)
Right side	14 (10.6)
Rectosigmoid	12 (9.1)
Left side	8 (6.1)
Transverse	5 (3.8)
**Sites of metastases**	
Liver	68 (42.8)
Lung	38 (23.9)
Others	4 (2.5)
No metastases	49 (30.8)
**Histopathology type**	
Well differentiated	41 (31.1)
Moderately differentiated	85 (64.4)
Poorly differentiated	6 (4.6)
**Treatment regimen**	
No irinotecan-based chemotherapy	66 (50.0)
Irinotecan-based chemotherapy	66 (50.0)

Supplement Table S1. Clinical characteristics of 132 colorectal cancer patients
